# Bilateral atypical insufficiency fractures of the proximal tibia and a unilateral distal femoral fracture associated with long-term intravenous bisphosphonate therapy: a case report

**DOI:** 10.1186/1752-1947-6-50

**Published:** 2012-02-06

**Authors:** Arjuna Mahinda Imbuldeniya, Natasha Jiwa, John Paul Murphy

**Affiliations:** 1 The North West London Hospitals NHS Trust, Northwick Park Hospital, Watford Road, Harrow, HA1 3UJ, UK

## Abstract

**Introduction:**

Atypical insufficiency fractures of the femur in patients on long-term bisphosphonate therapy have been well described in recent literature. The majority of cases are associated with minimal or no trauma and occur in the subtrochanteric or diaphyseal region.

**Case presentation:**

We describe the case of a 76-year-old British Caucasian woman who presented initially to an emergency department and then to her primary care physician with a long-standing history of bilateral knee pain after minor trauma. Plain radiographs showed subtle linear areas of sclerosis bilaterally in her proximal tibiae. Magnetic resonance imaging confirmed the presence of insufficiency fractures in these areas along with her left distal femur. There are very few reports of atypical insufficiency fractures involving the tibia in patients on long-term bisphosphonate therapy and this appears to be the only documented bilateral case involving the metaphyseal regions of the proximal tibia and distal femur.

**Conclusion:**

In addition to existing literature describing atypical fractures in the proximal femur and femoral shaft, there is a need for increased awareness that these fractures can also occur in other weight-bearing areas of the skeleton. All clinicians involved in the care of patients taking long-term bisphosphonates need to be aware of the growing association between new onset lower limb pain and atypical insufficiency fractures.

## Introduction

Atypical subtrochanteric and diaphyseal insufficiency fractures of the femur in association with long-term bisphosphonate use have been well described in recent literature [1-6]. The majority of cases are associated with the most commonly used bisphosphonate alendronate but may also be associated with other antiresorptive agents, corticosteroids and proton pump inhibitors [4,7].

By causing osteoclast apoptosis, bisphosphonates decrease bone resorption. This results in increased bone mineral density and a decreased risk of fracture during the first five years of administration [8]. Animal studies, however, demonstrate an accumulation of microdamage and a lack of effective remodeling within bone after bisphosphonate use, which has been shown to compromise its biomechanical properties [9,10]. In addition, a histomorphometric analysis of cancellous bone biopsy samples in nine patients sustaining spontaneous non-spinal fractures whilst on bisphosphonate therapy revealed markedly suppressed bone turnover [4]. It has also been proposed that long-term bisphosphonate use may lead to secondary mineralization producing more brittle bones [11].

We describe a case of atypical bilateral proximal tibial and unilateral distal femoral insufficiency fractures in an adult woman who had been receiving tri-monthly intravenous pamidronate treatment for approximately six years.

## Case presentation

A 76-year-old British Caucasian woman presented with a seven-month history of bilateral knee pain, caused initially by moving heavy furniture. Her symptoms were exacerbated on her left side after striking her knee against a parked car door five weeks prior to presentation. She could recall initial mild swelling which settled but no bruising. Her pain was worse when weight bearing, particularly on her left leg. An examination of her lower limbs was unremarkable except for tenderness over the medial proximal tibias bilaterally. She was initially diagnosed with cellulitis around her knee by the emergency department, though the C reactive protein was only mildly elevated at 23, and was prescribed 5 days oral flucloxacillin. Subsequent investigations for deep vein thrombosis by her primary care physician were negative and her symptoms persisted.

Her relevant medical history included seropositive erosive rheumatoid arthritis for over 30 years, osteomalacia and osteoporosis. Her relevant drug history included calcium 1200 mg/day, vitamin D3 800 units/day, prednisolone 5 mg/day, rabeprazole 20 mg/day, folic acid 10 mg once a week, intravenous methotrexate 15 mg once a week, and 30 mg intravenous pamidronate every three months. She had been taking prednisolone and methotrexate for seven years continuously. Blood tests revealed that her vitamin D, calcium, phosphate, parathyroid hormone and alkaline phosphatase levels were within normal limits.

Our patient had been treated with oral alendronate for three years but due to poor tolerance and compliance, her therapy was changed to intravenous pamidronate for the following six years. Measurement by dual-energy X-ray absorptiometry (DXA) was performed at her hip and lumbar vertebrae three years before bisphosphonate treatment was commenced and again three years after. Her T scores at her hip improved from -1.55 to -1.3 and at her lumber vertebrae from -2.86 to -2.5.

**Figure 1:**
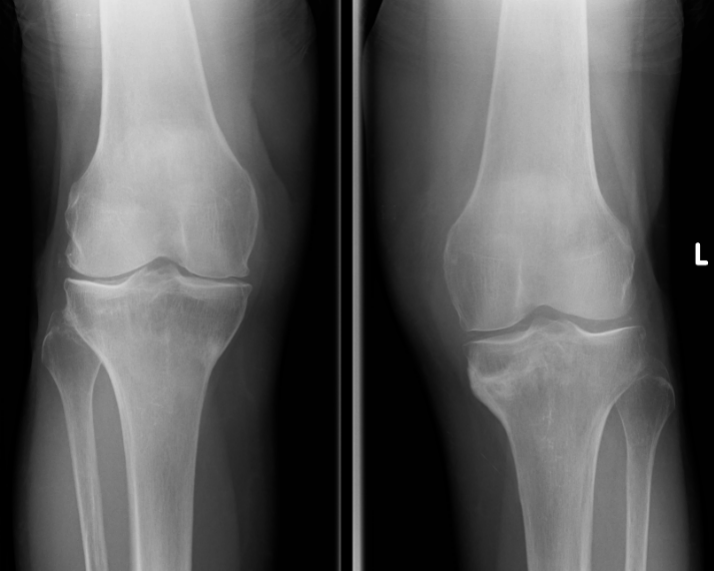
**Anteroposterior radiographs seven months after the initial symptoms, showing osteosclerotic change with a periosteal reaction at both medial proximal tibias**.

Her rheumatologists initially treated the knee pain with single intra-articular hydrocortisone injections. Subsequent plain radiographs of both her knees were reported by the radiologists as having linear areas of sclerosis involving the medial metaphyseal regions of both her proximal tibiae. The changes were reported as more marked on her left side associated with a periosteal reaction suggestive of either insufficiency or stress fractures. No radiographic changes were demonstrated around her left distal femur (Figure 1).

Our patient was referred to the orthopedic department where she stated her bilateral knee pains had now significantly improved. On examination, she was found to have a normal gait, mildly swollen knees bilaterally with tenderness over her left proximal medial tibia and a good equal range of knee, hip and ankle movement.

**Figure 2:**
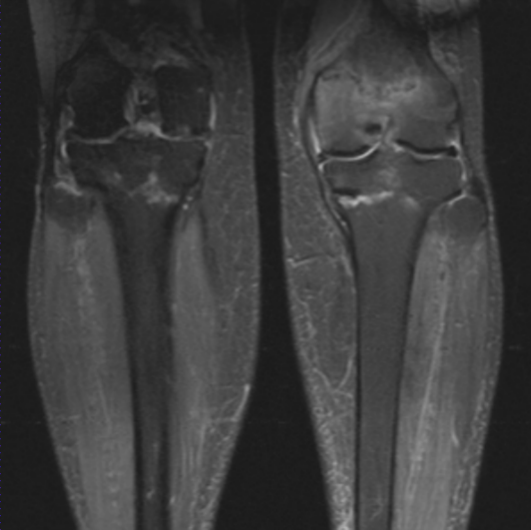
**Magnetic resonance imaging coronal short inversion time inversion recovery images show a linear signal change in both the proximal tibia and left distal femur with surrounding reactive change**.

Magnetic resonance imaging scans were organized and demonstrated extra-articular linear signal change on T1, T2 and short inversion time inversion recovery images with surrounding marrow edema associated with insufficiency or stress fractures at the level of the proximal tibiofibular joint within both tibiae. Her left knee also demonstrated a fracture line through her distal femur with associated edema more marked medially and centrally. Her left medial collateral ligament had a slightly increased signal in keeping with a recent sprain with marked edema in the suprapatellar fat pad and surrounding soft tissues. There was spotty bone marrow edema within her right distal femur, but no discrete linear fracture line was demonstrated (Figure 2).

Her treatment consisted of bilateral knee supports, rest and protected weight bearing with crutches as tolerated until her symptoms fully resolved within two months.

**Figure 3:**
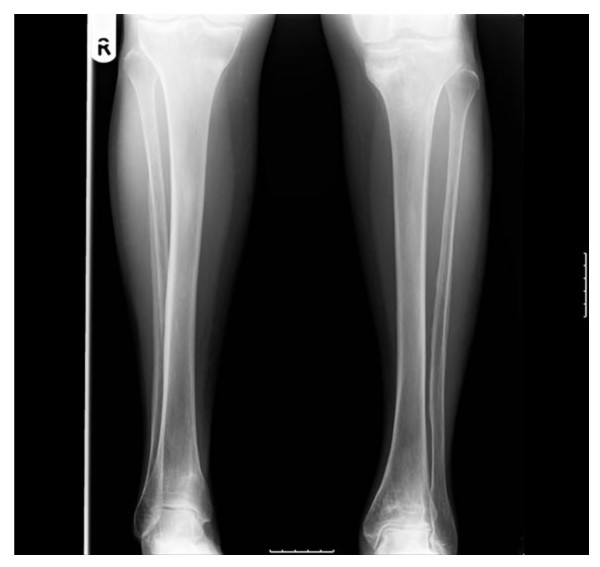
**Anteroposterior radiographs of both tibiae six months after diagnosis with resolution of symptoms and healed fractures**.

**Figure 4:**
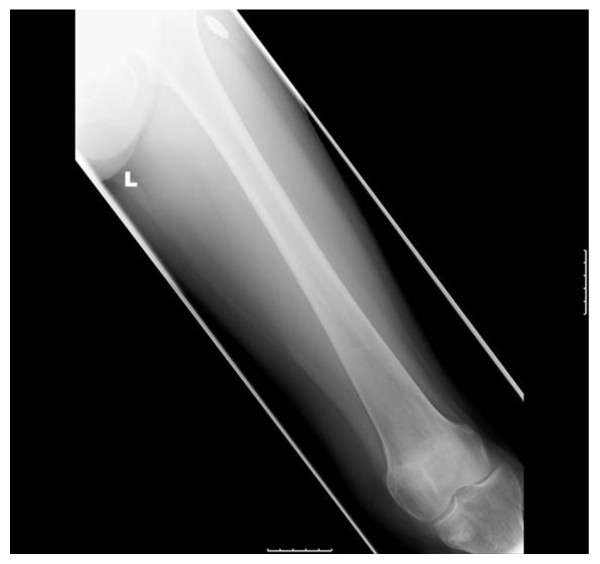
**Anteroposterior radiographs of the left femur six months after diagnosis with resolution of symptoms and healed fractures**.

**Figure 5:**
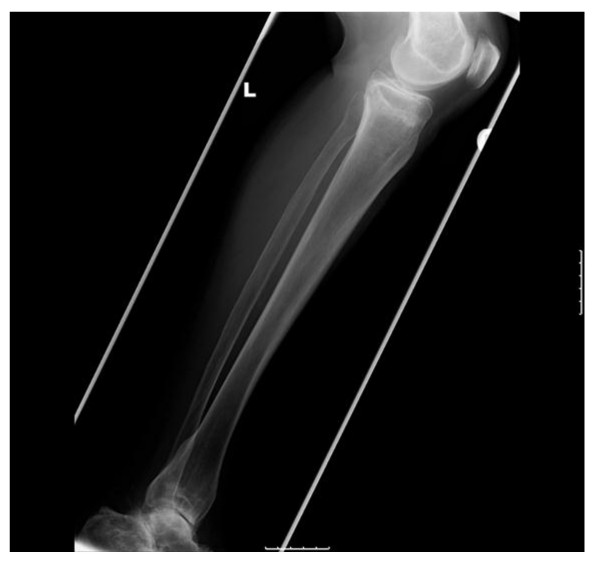
**Lateral radiograph of the left tibia six months after diagnosis with resolution of symptoms and healed fractures**.

**Figure 6:**
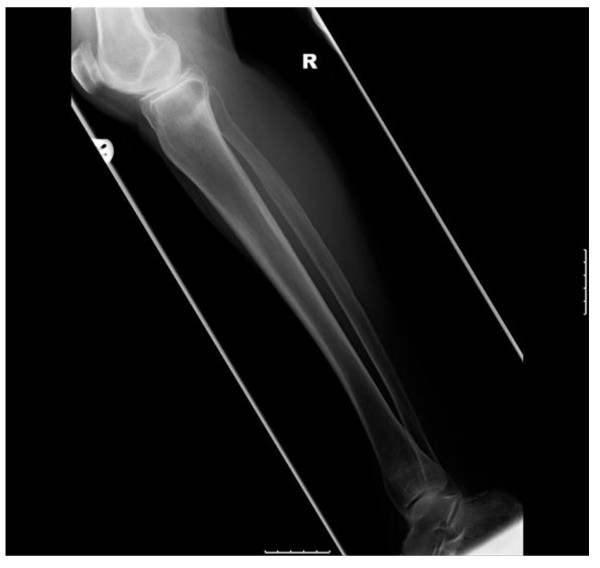
**Lateral radiograph of the right tibia six months after diagnosis with resolution of symptoms and healed fractures**.

**Figure 7:**
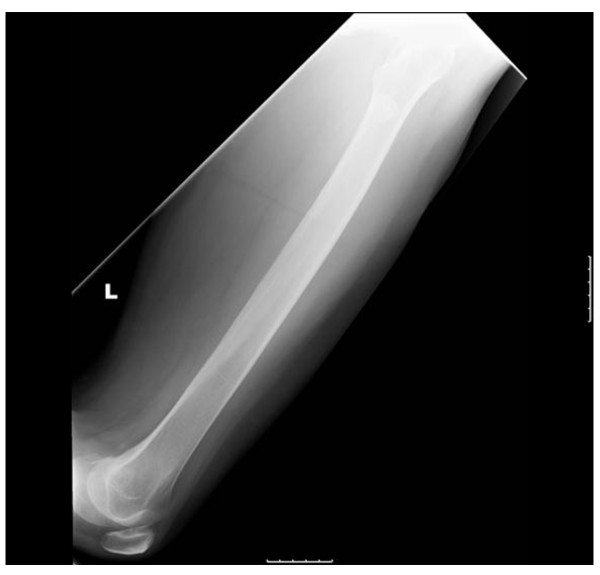
**Anteroposterior radiographs of the left distal femur six months after diagnosis with resolution of symptoms**.

Our patient was reviewed one year after presentation with up-to-date radiographs demonstrating healed fractures around both knees (Figures 3, 4, 5, 6 and 7). Unfortunately, though her knee symptoms were no longer an issue, she had sustained a right sided extracapsular femoral neck fracture six months previously after a mechanical fall. This fracture had been treated operatively with a dynamic hip screw and she was making good progress with rehabilitation and recovery, mobilizing at her last review without any walking aids. Her rheumatologists had stopped her intravenous pamidronate as a caution over the last six months in view of her multiple insufficiency fractures.

A follow up DXA scan four months previously showed no significant change in her bone mineral density from the last result. No other changes were made to her drug therapy during or after this period.

## Discussion

Long-term bisphosphonate therapy has recently been associated with atypical proximal metaphyseal and diaphyseal femoral insufficiency fractures in a number of case series and reports [1-4,6,7,12-14]. Large randomized trials, however, have not shown a significant increased risk or a causal relationship [12]. This case highlights what we believe are atypical bilateral tibial and unilateral femoral insufficiency fractures occurring in a patient on long-term bisphosphonate therapy. Previous reports of atypical tibial insufficiency fractures are few in number [15,16] and describe fractures of the tibial diaphysis as opposed to the proximal metaphyseal fractures seen in this case. Similarities of note between the cases are that one patient had rheumatoid arthritis [15], whilst the other patient was taking calcium and vitamin D supplements [16]. All the patients were fully active prior to injury and the fractures were associated with minimal trauma.

The long-term oral corticosteroids and proton pump inhibitors which our patient was taking have been recognized as possible causes for increased fracture risk [4,14,17]. An increased risk of both vertebral and nonvertebral fractures has been reported with dosages of prednisolone or equivalent as low as 2.5 mg to 7.5 mg daily, and this risk may relate more strongly to daily rather than to cumulative doses of glucocorticoids [18]. In a large meta-analysis, prior and current use of oral glucocorticoids increased the risk of any type of fracture, with no significant difference in relative risk between men and women [19].

There is also a link between methotrexate and insufficiency fractures, though in the context of rheumatoid arthritis this has not been as well established [20]. Studies have shown increased urinary and fecal excretion of calcium in methotrexate use, indicating enhanced osteoclastic bone resorption, and toxic effects on osteoblasts resulting in osteopathy. There have also been case reports of tibia fractures in patients on low dose to intermediate dose methotrexate therapy [21].

Cases in the literature of atypical fractures in patients on bisphosphonate therapy include those who were not taking any of the above mentioned agents and so a direct causal relationship has yet to be found [2,13].

Insufficiency fractures differ from stress fractures, in that the bone is abnormal in terms of mineralization, elasticity and ability to repair from injury, and so normal physiological activity can result in fracture [22]. The majority of documented cases have occurred in patients using alendronate, which is the most commonly used bisphosphonate. However, atypical insufficiency fractures have also been reported amongst users of ibandronate, risedronate, zoledronate and pamidronate [2,6,7,12,13,16,23]. In a child with osteogenesis imperfecta, prolonged high dose administration of intravenous pamidronate has also been associated with the development of osteopetrosis [11].

Histomorphometric analysis of bone biopsy samples in patients taking alendronate has previously revealed severe suppression of bone turnover [4]. Certainly the absence of this investigation in our described case is a limitation when attempting to establish an association between long-term bisphosphonate therapy and atypical insufficiency fractures. A bone biopsy could also have helped differentiate osteomalacia from osteoporosis as the underlying pathology.

It has been recommended that new onset thigh or hip pain in patients on long-term bisphosphonate therapy should be investigated with appropriate plain radiographs [1]. We would extend this recommendation to bone or joint pain in any weight-bearing area, with or without a history of low-energy trauma. This is important as prodromal pain has been frequently reported in other series [2] and was also present in our case. It should be noted, however, that it can take between two and six weeks from the onset of symptoms to detect any radiographic changes.

Looser's zones are associated most frequently with osteomalacia [24], which could be a cause for these fractures. They have sclerotic irregular margins and are often symmetrical with wide transverse lucencies traversing the bone, usually at right angles to the involved cortex. These changes are present in the plain radiographs but are fairly subtle (Figure 1). Although our patient takes supplementation for the treatment of osteomalacia, her blood test results suggest this has been well controlled for a number of years. The typically described radiographic findings in previous case series of atypical insufficiency fractures of the proximal metaphysis and diaphysis of the femur, those of cortical thickening and a transverse fracture pattern, were also seen in our case though there was no cortical spiking or beaking [13,15].

The well-known benefits of bisphosphonate treatment outweigh the relatively low risk of this rare group of atypical fractures. It also appears that, despite continuing bisphosphonate therapy, the majority of patients who sustain a fracture go on to make a full recovery within the expected timeframe. Further research is needed to provide guidance as to whether bisphosphonate therapy should be withheld whilst a patient is recovering from a fracture. More research is also needed to help identify any risk factors common to this subgroup of at-risk patients [14].

## Conclusion

The above case highlights that atypical insufficiency fractures can also occur in other weight-bearing areas of the skeleton, such as the distal femur and proximal tibia. A high index of suspicion is recommended for early diagnosis and prompt treatment amongst all clinicians involved in the treatment of patients taking long-term bisphosphonates. This is particularly important as such patients are commonly taking other medications which have also been implicated in increasing the risk of fracture.

## Consent

Written informed consent was obtained from the patient for publication of this case report and any accompanying images. A copy of the written consent is available for review by the Editor-in-Chief of this journal.

## Competing interests

The authors declare that they have no competing interests.

## Authors' contributions

AI prepared the manuscript, revised it critically for important intellectual content and performed a review of the literature. NJ acquired data and helped prepare the manuscript. JM managed the patient's clinical care. All authors read and approved the final manuscript for publication.
